# Mental Health Applications for Primary and Secondary Prevention of Common Mental Disorders: Attitudes of German Employees

**DOI:** 10.3389/fpsyt.2021.508622

**Published:** 2021-05-04

**Authors:** Martina Michaelis, Stephanie Burgess, Florian Junne, Eva Rothermund, Harald Gündel, Stephan Zipfel, Markus Wolf, Monika A. Rieger

**Affiliations:** ^1^Institute of Occupational and Social Medicine and Health Services Research, University Hospital Tübingen, Tübingen, Germany; ^2^Research Centre for Occupational and Social Medicine (FFAS), Freiburg, Germany; ^3^Department of Internal Medicine VI, Psychosomatic Medicine and Psychotherapy, University Hospital Tübingen, Tübingen, Germany; ^4^Department for Psychosomatic Medicine and Psychotherapy, University Hospital, Otto von Guericke University Magdeburg, Magdeburg, Germany; ^5^Department of Psychosomatic Medicine and Psychotherapy, Ulm University Medical Center/Leadership Personality Centre, Ulm University, Ulm, Germany; ^6^Center for Psychotherapy Research, University Hospital Heidelberg, Heidelberg, Germany; ^7^Department of Psychology, University of Zurich, Zurich, Switzerland

**Keywords:** e-mental health, mental health apps, survey, employees, attitudes, mobile health, common mental disorders

## Abstract

**Background:** Web-based and mobile mental health applications for the prevention and treatment of common mental disorders (CMDs) are on the rise. Under certain circumstances they have proved to be effective for a range of conditions (e.g., depression).

**Objective:** There is not sufficient evidence regarding the benefits and barriers especially for mobile phone apps and for programs in the field of primary prevention. Studies on the acceptance of potential users of mental health apps yielded mixed outcomes. In a large survey we investigated the attitudes of employees toward mental health apps and various traditional mental health services. Our main research question in this contribution focuses on the acceptance of apps compared to other measures and the moderating influence of individual characteristics.

**Methods:** The standardized survey was completed by members of an online access panel with different job types. A set of 33 self-developed items, including three questions on e-health, captured the perceived relevance of prevention at the (A) occupational, (B) individual, and (C) societal level. On the basis of an exploratory factor analysis, mean scores for mapping seven (sub-)dimensions were constructed and compared using the Wilcoxon test. The influence of potential predictors was analyzed in linear regression models.

**Results:** The data of 610 respondents were analyzed (response rate 75%). Support from mental health applications was rated significantly less important compared to all other dimensions at the levels (A) to (C). Respondents were more likely to use mental health apps if they felt literate with electronic devices, perceived a high relevance of work-related demands as causal factors for CMDs, stated they would be ashamed of having a CMD, and would be willing to begin psychotherapy if recommended.

**Discussion and Conclusions:** The results confirm the critical attitudes of potential mental health app users found in other studies. Since users with a negative attitude toward e-health might have a higher risk for dropout and non-adherence as well as lower intervention effects, well-designed educational strategies should be carried out beforehand.

## Introduction

In the working population common mental disorders (CMDs), such as depression and anxiety disorders, are becoming increasingly prevalent (1–4). Effective prevention strategies are needed, which should consider a broader spectrum of factors beyond workplace factors. In addition, electronic mental health applications (e-mental health apps) and mobile phone applications (m-mental health apps) for primary and secondary prevention are becoming increasingly available. However, to date, there is not enough evidence regarding the reach, benefits, barriers, and possible harm of using such apps (5–7).

Guided online interventions for preventing and treating CMDs and blended treatment, that is, the combination of face-to-face and digital approaches, may on the one hand be considered effective for people who are incapable or not willing to use other services (8). These services can be compensatory for long waiting periods for therapy or increase access to, as well as the reach and sustainability of traditional services for people with increasing problems (9). On the other hand, there is evidence for low adherence, high attrition, and ambivalent attitudes of potential users of e-mental health services (10–13) and m-mental health features (14). In our study “PHOEBE II,” we investigated the attributed relevance of different prevention approaches from employees' points-of-view (15, 16). The survey followed partly an earlier similar investigation among healthcare providers and human resource managers in the year 2014 (“PHOEBE I” study (17–19). In the current employees' survey, we added items on e-mental and m-mental health. Our research questions were:

- What do German employees with a broad range of professions think about the relevance of using e-mental health applications to prevent and treat CMDs?
- Which value do these attitudes have compared to diverse other, more traditional prevention measures on an individual, workplace, and societal level?
- Is there a difference between attitudes Toward e-mental health and m-mental health applications?
- Which individual factors and opinions influence the respondents' attitudes?

## Methods

### Study Design and Frame, Participants

For the cross-sectional survey in the year 2016, we targeted access panel members of a commercial market and opinion research institute (*Research Now SSI – Dynata*) including almost all economic sectors. Inclusion criteria concerned persons working in dependent employment and aged between 18 and 64 years. Three categories of job types were stratified prior to recruitment to meet the requirement of diverse professions (20, 21):

- Blue-collar workers: Manufacturing/processing/craft occupations.
- Gray-collar workers: Care, support, and medical assistance occupations, service occupations in the areas of facility management (caretakers, building cleaning, and cleaning activities, security services), warehouse/logistics/transport, catering/hotel industry, trade).
- White-collar workers: Office, social, and educational professions.

Participants were rewarded with small incentives (shopping vouchers). Non-target participants, refusers, and study dropouts were replaced by randomly selected new participants from the same job types. Incomplete data sets were avoided through completion control implemented in the online tool.

### Instruments and Variables

The survey included a selection of self-constructed items which was partly based on a previous study (“PHOEBE I”) (17). Its usability was pretested with 11 employees. The relevance of preventing CMDs was assessed using 17 items with three questions covering workplace prevention issues, five items related to societal prevention, and 11 items covering individual prevention (4-point Likert-scaled from 1 “not relevant at all” to 4 “very relevant”). With regard to individual prevention, the overall question was: “Each individual can do something for him- or herself to reduce the risk of CMD or its respective consequences. In your opinion, how important are the following activities?”

Out of 11 individual prevention items, four items were used as an index on the “Use of mental health applications as a strategy for the prevention of CMDs” and to calculate mean scores. To confirm structural validity and internal consistency of this index, an exploratory factor analysis was carried out and Cronbach's α was assessed. The structural validity was confirmed by the respective two items addressing e-mental applications and one item covering m-health applications, with factor loadings of 0.8. The items were expressed as follows (for details see Supplementary Table 1):

- Use of online self-help programs (which can be worked through independently).
- Utilization of professional online-Counseling (e.g., email or chat with a coach/a psychotherapist).
- Use of applications on the mobile phone (e.g., support of such an application in the case of respective problems).

A fourth item with multiple low factor loadings was manually attributed to “Mental health applications: Expanding one's knowledge about CMDs by reading”.

Other subdimensions confirmed in the respective prevention areas by factor analysis are listed below.

- For the area of workplace prevention: “Design of work and qualification,” “Training and coaching for managers and employees,” and “Behavioral prevention offers for employees”.
- For the area of individual prevention: “Support from specialists” (actively seeking psychotherapy, consulting a psychological counseling center/a general practitioner/an occupational health physician, participation in a statutory health insurance course, such as stress management), and “Support in private life” (asking family or friends for support, leading a healthy lifestyle).
- “Societal prevention activities” (one factor-solution).

To assess the association of predictors with the assessed relevance of mental health applications (subscore, see research question 4), the following variables were analyzed exploratively in a linear regression analysis:

- job type, age, gender, education,
- experience with CMDs in one's own social environment,
- own experience with CMDs,
- health-seeking behavior (global item about the willingness to begin a recommended psychotherapy in the case of one's own CMD),
- fear of stigma (shame in the theoretical case of one's own CMD, global item),
- e-health literacy (global item), and
- perceived relevance of work-related causes of CMD (mean score of 15 self-constructed items covering work-related demands: work content, work organization, and interpersonal relations and leadership)

For additional details see Burgess et al. (16), the study protocol (15) and Supplementary Tables 1, 2.

### Statistical Analysis

Dropout and non-responder analyses were undertaken, controlling for job type and systematic termination at potentially critical items.

To identify relevance rankings of dimensions (research questions 2 and 3), differences between index scores were analyzed with the non-parametric Wilcoxon test using IBM SPSS, version 23. The respective effect size “r” was calculated by z/root(cases) following the recommendations of Cohen (22) and classified as < 0.3, < 0.5, and ≥ 0.5, indicating a low, moderate, and high effect size (23).

As a result of the association between predictors and the assessed relevance of mental health applications, a parsimonious linear regression model to minimize suppressor effects (24) will be presented after excluding all non-significant variables by stepwise backward selection (p_(out)_ = 0.051). Model effect sizes are defined by means of R^2^ [> 0.02 low, > 0.15 moderate, > 0.35 strong (25)].

## Results

### Sample Description

Overall, 1,104 individuals were contacted and 610 participants were analyzed (net response rate 75.4%). The distribution had more white-collar than blue- and gray-collar workers (*n* = 248, 41% vs. *n* = 193 and 169; 31 and 28%). Nine percent study dropouts during the survey showed no job type- or item-related sample bias concerning age and gender. The sample characteristics can be derived from Table 1. Considering age and gender, the distribution was similar to the German employee population (26). With the high proportion of respondents who completed the German “Hauptschule” (lower secondary school) (61%), respondents were overrepresented compared with the German general population with about 30% (27).

**Table 1:** Sample characteristics (*n* = 610).

	**Mean (SD)**	**Percentage (*n*)**
Age	42.0 (12.7)	
Gender (female)		44.3 (270)
Education (German…):		
Hauptschule (lower secondary school)/no degree		61.1 (373)
Realschule (secondary school)		13.9 (85)
Gymnasium (high school or equivalent general qualification for university entrance)		24.9 (152)
Experiences with CMDs in one's own social environment (yes)		55.1 (336)
Own experience with CMDs (yes)		49.8 (304)
Health-seeking behavior (global item about the willingness to begin a recommended psychotherapy in the case of one's own CMD)^a^	3.2 (0.8)	
Fear of stigma (feeling of shame in the case of one's own CMD, global item)^b^	4.3 (2.4)	
Literacy with electronic devices (global item)^c^	2.5 (0.6)	
Perceived relevance of work-related demands with regard to CMDs in employees (total mean score)^d^	3.1 (0.5)	

*Global items, Likert scale:*

a *1 “not at all,” 2 “probably no,” 3 “probably yes” 4 “yes, definitely”;*

b *from 1 “not at all” to 9 “strongly”;*

c *from 1 “not good at all” to 5 “very good”;*

d *from 1 “very irrelevant” to 4 “very relevant”*.

### Attitudes Toward the Relevance of Mental Health Applications in Preventing CMDs

**Figure 1:**
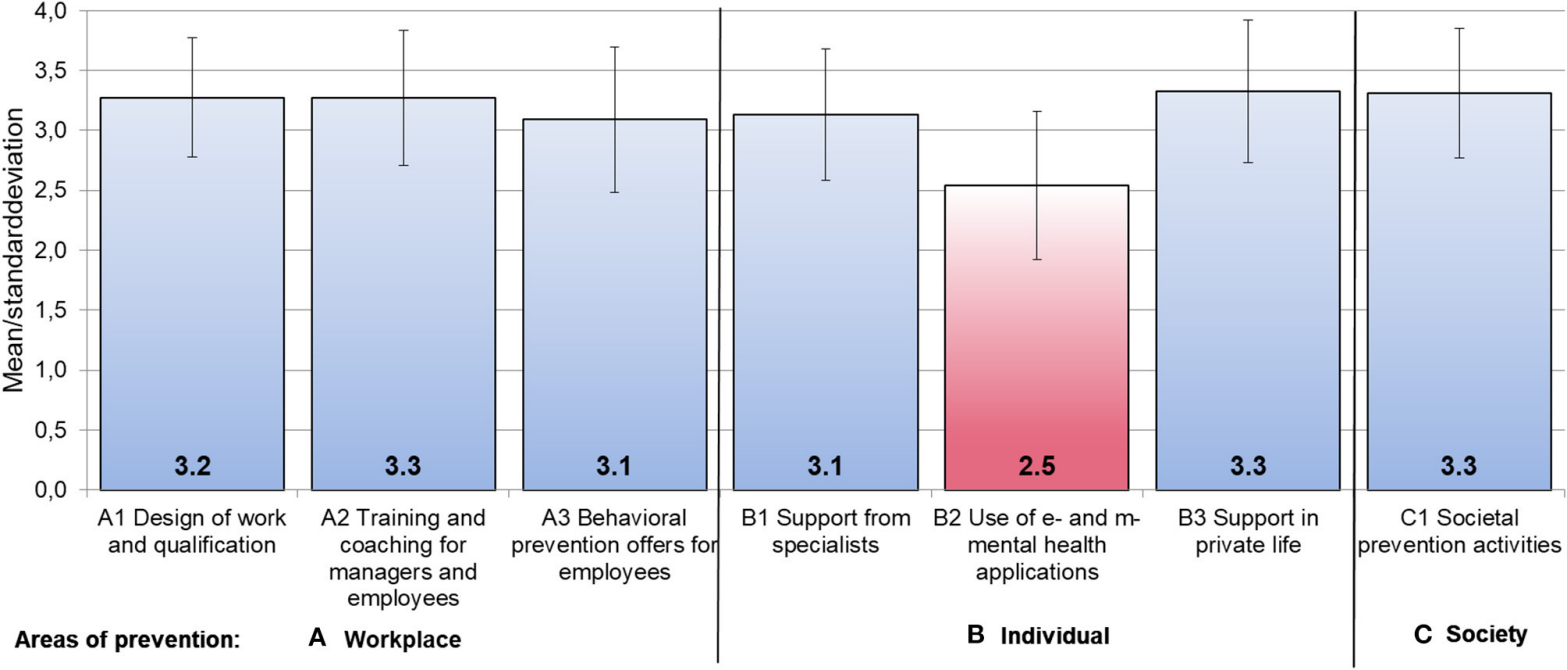
Attributed relevance (M, SD) of diverse prevention strategies at **(A)** the workplace, for **(B)** the individual, and in **(C)** social areas. Mean scores based on 4-point Likert scale from 1 “not relevant at all” to 4 “very relevant”; dimensions resulted from exploratory factor analysis.

To answer to the main outcome (Research Question 1 in the Introduction Section), the suggested strategies in the three prevention areas (workplace/individual/societal) were on average assessed as “rather” to “very” relevant (score means ranged from M = 3.1, SD = 0.61, to M = 3.3, SD = 0.56, where 4 = very relevant). There was one exception: Mental health applications were rated significantly lower (M = 2.5, SD = 0.6; Research Question 2; see Figure 1) than all other strategies (p_(Wilc)_ < 0.001; *r* = 0.66 to 0.78).

**Figure 2:**
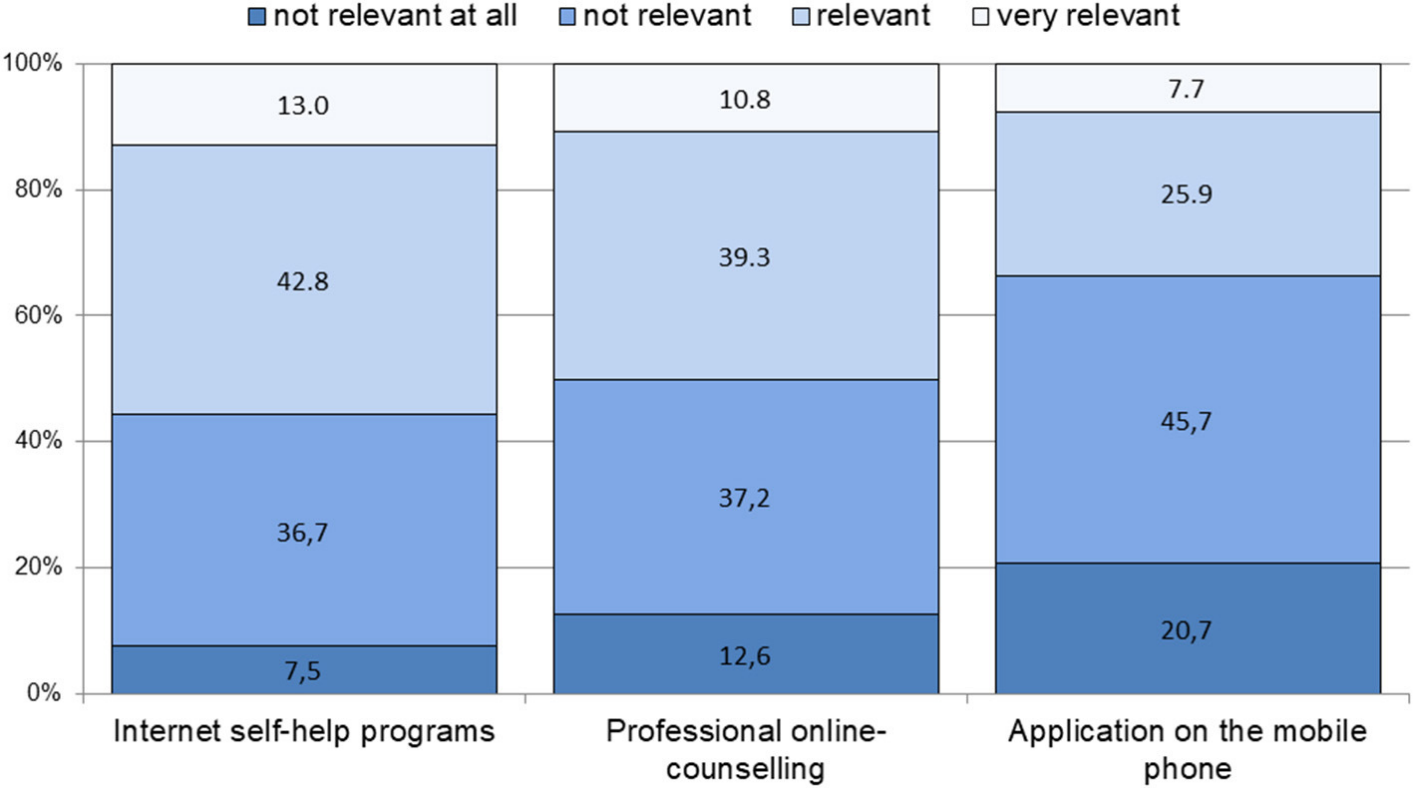
Use of mental health applications as a prevention strategy to prevent common mental disorders: attitudes toward relevance. Single items: frequency of answers on a 4-point Likert scale from 1 “not relevant at all” to 4 “very relevant”.

On item level, the relevance of mobile applications was rated significantly lower than web-based self-help programs or professional online consulting (*p* = 0.000, r_(Wilc)_ = 0.46 and 0.36, respectively; see Research Question 3 and Figure 2).

### Predictors for Attitudes

To answer to Research Question 4, a multiple linear regression analysis showed that respondents rated the relevance of e-mental health applications (mean score) as a prevention strategy higher, the more relevant they assessed work demands as a cause for developing a CMD, the more competent they rated themselves in using electronic devices, and the more likely they were to follow recommendations to begin psychotherapy in case they had a CMD (see Table 2). Further, the smallest effect was seen in employees who showed higher acceptance for mental health applications and would feel ashamed if they had a CMD. No significant associations were found for the variables job type, age, gender, education, experience with CMDs in one's own social environment, or own experience of CMDs.

**Table 2:** Predictors for the attributed relevance of using mental health applications as a prevention strategy for CMDs (mean score).

	**B**	**SE**	**Beta**	***P***
(Constant)	1.31	0.16		0.000
Relevance of work demands for developing a CMD^a^	0.27	0.05	0.22	0.000
Literacy with electronic devices^b^	0.20	0.06	0.12	0.001
Willingness to begin a recommended psychotherapy in the case of one's own CMD^c^	0.10	0.03	0.13	0.003
Shame in the case of one's own CMD^d^	0.02	0.01	0.09	0.019

*Results of the parsimonious linear regression model; model R^2^ = 0.11 (backward elimination of variables). B, regression coefficient; Beta, standardized regression coefficient; CMD, common mental disorder; p, level of significance*.

a *Mean score from 1 “very irrelevant” to 4 “very relevant”*.

b *Global item from 1 “not good at all” to 5 “very good”*.

c *Global item: “not at all,” 2 “probably no,” 3 “probably yes” 4 “yes, definitely”*.

d *Global item from 1 “not at all” to 9 “strongly”*.

## Discussion

In our survey of 610 employees with a broad range of professions we assessed the relevance of and attitudes toward e- and m-mental health applications for the primary and secondary prevention of mental health disorders. In a second step, we compared these values to more traditional prevention measures on an individual, workplace, and societal level. We pointed out the difference between attitudes toward e-mental health and m-mental health applications and evaluated individual factors and opinions as moderator variables of the respondents' attitudes.

Our results show that acceptance was low, compared to more traditional measures or interventions. This was true for online consultations with a mental health professional and online self-help programs, and even more for mobile devices applications (m-health). Similar constrained and reserved attitudes of potential users of mental health applications were also found by other authors (10, 13, 27–30).

Reasons for the low acceptance can be seen in preferences for face-to-face interventions (31), but also in a lack of knowledge and information about respective programs, their quality, and their effectiveness. In Germany, some statutory health insurance companies currently offer e-mental and m-mental health applications to their members. In addition, some programs can be obtained directly from commercial providers, or are available for free on the Internet, or in app stores. In most cases, however, access to e-mental and m-mental health applications is limited to research projects which are not accessible to the public (7, 32, 33).

Given the current diversity of such programs (7) and the substantiated lack of awareness among the general population (11), the low level of acceptance is not surprising. In particular, when most people are unlikely to have any experience with e-mental health programs. There is preliminary evidence that a respective intervention might increase the patients' awareness and acceptance (34). However, there are other issues, such as data protection concerns, that must be seen as barriers (7, 27), whereas convenient access can be seen as a facilitating factor (27).

The attitudes of the respondents were not associated with age, gender, education, or job type in our large sample of German employees. Concerning the first three predictors, similar results were found in other studies (10, 13, 22, 35). Other studies that focused on the use of digital technologies also showed similar results (35, 36). An assumed higher professional IT- experience of white-collar workers compared to gray- and blue-collar workers, thus, was not reflected in our data. The rough categorization into three job types, however, was too limited to draw up final conclusions. The predictor “literacy with electronic devices” might be more meaningful.

The respondents' perceptions of work demands as causative factors for CMDs were the strongest predictor for a general openness to e-mental health interventions in our study. Similar results were found by Apolinario-Hagen et al. (10), with a correlation between a positive evaluation of web-based therapies and self-reported distress at the time of study participation. Based on these findings, specific target groups for dissemination and information about respective programs could be identified in economic branches with known high mental work demands. As diverse reviews and meta-analysis in recent years could show, occupational e-mental health interventions can evidently improve workers' mental health and work effectiveness, although the effects are small, at least short-term (36–40). As Albrecht and Jan stated in the German CHARISMHA study, hardly any meaningful data are available for a comprehensive evaluation of the long-term effects of apps that focus on primary prevention in general (41).

The impact of a person's own experience and literacy with electronic devices on the acceptance of mental health applications has been found in other studies (32, 33), especially among participants who would be affected by CMDs (41). So called “e-preferers” can therefore be regarded as a particularly accessible target group. To increase effectiveness and adherence, future interventions should assess individual skills, treatment preferences, and attitudes toward e-mental health to offer adequate and well-accepted services (42).

As already mentioned, there is an ever-increasing number of e- and m-mental health applications (43, 44) partly available on specific online platforms (e.g., http://myhealthapps.net or https://www.appbrain.com) or general platforms as the German app stores of Google Play and iTunes.

However, a common definition of quality criteria (44) as well as sufficient evidence for effectiveness is still lacking (45), especially in the primary prevention sector. In samples like ours involving employees, the evidence is mixed regarding the effectiveness of e-mental health programs. In a recent Cochrane review, Kuster et al. found very low evidence with conflicting results, when comparing the effectiveness of computer-based stress management interventions with in-person stress management interventions (6). Another recent meta-analysis, however, stated that “web- and computer-based stress-management interventions can be effective and have the potential to reduce stress-related mental health problems on a large scale” (46).

The potential of mobile technology to enhance healthcare service delivery (m-mental health) and the evidence for its acceptability, feasibility, and efficacy is increasing (32). However, possible benefits might not yet meet the knowledge of potential users. Besides the need for more research on the implementation and integration of respective interventions, there is a need for more and detailed information. So far, resources to assess high-quality apps and interventions in order to compile them in lists are availiable (47–50). We assume that also mental health professionals are often not aware of these options to help make informed decisions or to guide employees with mental health problems to find a suitable e-mental health service among those who are open to use such a service and have the digital literacy to use it.

### Limitations and Strength of the Study

It should be kept in mind that e-mental and m-mental health is a fast moving field. While our survey was conducted in 2016, in the meanwhile a number of German national regulations have changed, for instance with the introduction of the *Digital Health Care Act* (2019) (51), which might have had an indirect impact on employees' and also on professionals' attitudes (52). Also, at the time of data collection, there were hardly any activities in Germany in the direction of digital health promotion in the primary prevention sector. Future surveys should thus reassess employees' attitudes and address potential factors influencing attitudes toward digital health today.

Recruitment through commercial online access panels limits the representativeness of the working population assessed in our study. This is due to the fact that most of our employees included in the sample had “worker” positions and a lower educational level compared to the general German population. Thus, the number of employees in management positions is lower in our sample (16). However, through this approach we were able to gain insights into a relevant cohort of employees beyond higher educational level who are able to cope with mental health applications.

Further, we were able to reach a broad range of professions, which is generally difficult to achieve using surveys conducted in companies. Evaluation of three global items is of course limited. For future studies it would be advisable to put more focus on theoretical acceptance, including promoting factors.

With more than half of the respondents reporting own prior experiences with a CMD, our sample might be somewhat biased toward traditional services. This could explain the higher acceptance rates in these domains. A critical discussion of strengths and limitations after surveying an online access panel can be found in Burgess et al. (53).

## Conclusions

Therapeutic assistance seems to be essential for the acceptability of e-mental and m-mental health programs. This should be considered when developing respective applications, even in the secondary prevention sector, where effectiveness is well-researched. Especially m-mental health applications are on the rise and might also be useful in primary prevention, specifically in the workplace setting.

Although certainly the digitization push since the onset of the SARS-CoV-2- pandemic has increased the availability and acceptance of e- and m-mental health (54), new strategies in order to increase acceptability within the target groups are required. In addition to global information strategies of statutory health and accident insurances, credible, trusted, and appropriately trained health professionals could host and provide respective evidence-based services. In future research, actual psychological work-related demands and distress of a person should be investigated as influencing factors for the attributed relevance and acceptance of mental health applications.

## Data Availability Statement

The datasets generated for this study are available on request to the corresponding author.

## Author Contributions

MR, MM, HG, SZ, ER, and FJ conceived and designed the study. All persons named were substantially involved in data acquisition and interpretation. MW provided e-mental health expertise and was involved in the conceptualization and operationalization of the questionnaire. SB and MM performed the statistical analyses. MM and MR drafted the manuscript. ER, FJ, SZ, HG, MW, and SB critically revised it. All authors gave final approval of the version to be published and agreed to be accountable for all aspects of the work.

## Conflict of Interest

The authors declare that the research was conducted in the absence of any commercial or financial relationships that could be construed as a potential conflict of interest.
