# Supplementary material for: Mental Health Applications for Primary and Secondary Prevention of Common Mental Disorders: Attitudes of German Employees
Source: Front Psychiatry. 2021 May 4;12:508622. doi: 10.3389/fpsyt.2021.508622 (PMC8130826; doi:10.3389/fpsyt.2021.508622)
Supplement: Supplementary file 1 [file Table_1.DOCX]

**Supplementary Material**

**Table S1** Survey items and operationalization: Outcome and predictors for regression analysis (attitudes/perceptions and self-perceptions)

| **Area** | **Items and operationalization** | **Number of items/scaling** |
| --- | --- | --- |
| **Outcome** |  |  |
| Perceived use of mental health applications as a prevention strategy to avoid CMDs: attitudes towards relevance | In case of having mental health problems: How likely would you (also) use the following electronically supported services?   - Online self-help programs (which can be worked through independently) - Professional online-counseling (e.g., via email or chat with a coach/psychotherapist) - Applications on the mobile phone | 3 items, 4-point Likert scale (certainly no, rather no, rather yes, certainly yes) |
| **Predictors: Attitudes and perceptions** | |  |
| Relevance of work demands for developing a CMD | In your opinion, how significant are the following aspects in working life for the development of mental disorders?  Factors with relevance confirmed by occupational science and aspects addressed in the strategy of *the Joint Occupational Safety and Health Strategy*: Work-related requirements, work content, work organization, work environment, social relationships^(1)^, formulations aligned with COPSOQ constructs^(2)^ | 14 items: 4-point Likert scale (not relevant at all, rather not relevant, rather relevant, very relevant) |
| **Predictors: Self-attributions** | |  |
| Fear of stigma (shame in the theoretical case of one’s own CMD) | To what extent would you be ashamed of having a mental disorder? | 1 (global) item, polarity profile from 1-9 (shame not at all - very strongly) ^(3)^ |
| Health-seeking behavior (willingness to begin a recommended psychotherapy) | I would be willing to start psychotherapy if it was recommended for the treatment of a CMD | 1 global item, 4-point Likert scale (certainly not, rather not, rather yes, certainly yes) ^(3)^ |

Legend of abbreviations: CMD= Common mental disorder; COPSOQ= Copenhagen Psychosocial Questionnaire

^(1)^ German Joint Occupational Safety and Health Strategy (Gemeinsame Deutsche Arbeitsschutzstrategie, GDA) 2014. Available from <http://www.gda-portal.de>

^(2)^ Items analogous to early COPSOQ subscales: quantitative demands, qualitative demands, leadership behavior, emotional work demands, influence and development opportunities at work, work-privacy conflict, social relationships at work [1].

Items analogous to later COPSOQ constructs/adaptations: work environment [2], work processes [3], working time arrangements [4], trust and justice [5] (here in the sense of 'leadership culture'), self-constructed item 'Communication culture in the team/in the company'.

^(3)^ Modified following Rüsch et al. [6]

**Table S2** Questionnaire operationalization: Predictors for regression analysis (individual characteristics)

| **Area** | **Items and operationalization** | **Number of items/scaling** |
| --- | --- | --- |
| Sociodemographic characteristics | Age, gender, education (highest school or vocational qualification) ^(1)^ | 3 items: metric, dichotomous, categorical |
| Job type | White-, grey- and blue-collar workers | 1 item (classified by occupation) |
| Literacy with electronic devices | How often do you use a computer, tablet or smartphone for the following activities?   - Email - Information search on the Internet - Game software ^(2)^ | 3 items, 4-point Likert-scaled (never - rarely - occasionally - frequently) |
| Experience with own mental disorder | Do you have any experience with CMDs? | 1 global item: categorical (yes, no) |
| Experience with mental disorder in own living environment | Have you ever witnessed a CMD in friends, relatives or at your workplace? | 1 global item: categorical (yes, no, not sure) |

Legend of abbreviations: CMD= Common mental disorder

^(1)^ Classification of education by combining school qualification and vocational qualification according to the CASMIN classification (Comparative Analysis of Social Mobility in Industrial Nations), analogous to the health monitoring of the Robert Koch Institute, GEDA, <http://www.rki.de/DE/Content/Gesundheitsmonitoring/Studien/Geda/Geda_node.html>) [7]

^(2)^ Permitted withdrawal from the evaluation instrument in the project 'Guidebook for families with children having special disease presented by an app', in German [8]

**References for Tables S1 and S2**

1. Nübling, M., Hasselhorn, H.-M. (2010a): The Copenhagen Psychosocial Questionnaire in Germany: From the validation of the instrument to the formation of a job-specific database of psychosocial factors at work. Scand. J. Public Health 38(3),120-24. doi: 10.1177/1403494809353652
2. BMAS (2015): Desired and experienced quality of work. Final report of the representative survey commissioned by the Federal Ministry of Labor and Social Affairs (BMAS). Research Report 456. Berlin, Bonn, Münster/ Germany. Availiable on <https://www.bmas.de/DE/Service/Publikationen/Forschungsberichte/forschungsbericht-fb-456.html>
3. BAuA (2014): Risk assessment of mental stress – experiences and recommendations 1^st^ edition. Berlin/Germany: Federal Institute for Occupational Safety and Health (BAuA). Availiable on <https://www.baua.de/DE/Angebote/Publikationen/Fachbuecher/Gefaehrdungsbeurteilung2.html>
4. Nübling, M., Vomstein, M., Schmidt, S.G., Gregersen, S, Dulon, M., Nienhaus, A. (2010b): Psychosocial work load and stress in the geriatric care. BMC Public Health 10,428. doi: 10.1186/1471-2458-10-428
5. Pejtersen, J.H., Kristensen, T.S., Borg, V., Bjorner, J.B. (2010): The second version of the Copenhagen Psychosocial Questionnaire. Scand. J. Public Health; 38(3 Suppl), 8-24. doi: 10.1177/1403494809349858
6. Rüsch, N., Müller, M., Ajdacic-Gross, V., Rodgers S., Corrigan, P.W., Rössler W. (2014): Shame, perceived knowledge and satisfaction associated with mental health as predictors of attitude patterns towards help-seeking. Epidemiol. Psychiatr. Sci. 23(2), 177-87. doi: 10.1017/S204579601300036X
7. Lampert, T., Kroll, L.E., Müters, S., Stolzenberg, H. (2013): Measurement of socioeconomic status in the study 'Health in Germany actually (GEDA). Bundesgesundheitsblatt Gesundheitsforschung Gesundheitsschutz 56(1), 131-43. doi: 10.1007/s00103-012-1583-3
8. Braun, M., Kirschner, J., Vach, W., Boeker, M. (2014): Evaluation of a guidebook application for Duchenne muscular dystrophy. GMS German Medical Science, 2014 (online): 266 (59th . GMDS Annual Congress, Göttingen/Germany, 7^th^ to 10^th^ September 2014).
